# The association of pretreatment thrombocytosis with prognosis and clinicopathological significance in cervical cancer: a systematic review and meta-analysis

**DOI:** 10.18632/oncotarget.15358

**Published:** 2017-02-15

**Authors:** Juan Cheng, Zhi Zeng, Qingjian Ye, Yu Zhang, Ronghua Yan, Changyan Liang, Jia Wang, Mengxiong Li, Mixuan Yi

**Affiliations:** ^1^ Department of Gynecology, The Third Affiliated Hospital of Sun Yat-sen University, Guangzhou 510630, Guangdong Province, P.R. China; ^2^ Department of Radiology, The Third Affiliated Hospital of Sun Yat-sen University, Guangzhou 510630, Guangdong Province, P.R. China; ^3^ Reproductive Medicine Center, The Sixth Affiliated Hospital of Sun Yat-sen University, Guangzhou 510655, Guangdong Province, P.R. China; ^4^ Department of nephrology, The Second Xiangya Hospital, Central South University, Changsha 410001, Hunan Province, P.R. China

**Keywords:** inflammation, thrombocytosis, cervical cancer, prognosis, meta-analysis

## Abstract

Previous studies reported inconsistent findings about the relationship between pretreatment thrombocytosis and survival in patients with cervical cancer. This study aimed to evaluate the prognostic significance of thrombocytosis in cervical cancer. We searched databases to identify relevant articles. Pooled hazard ratios (HRs), odds ratios (ORs), and 95% confidence intervals (CIs) were calculated. Fourteen studies including 3,394 patients were eligible for the meta-analysis. Overall, an elevated platelet count was significantly associated with inferior overall survival (OS, hazard ratio [HR]: 1.66, 95% confidence interval [CI]: 1.42–1.95, *P* < 0.001) and recurrence-free survival (RFS, HR: 1.67, 95% CI: 1.15–2.42, *P* = 0.007) but not progression-free survival (PFS, HR: 1.21, 95% CI: 0.89–1.64; *P* = 0.235). The results were similar for low stage patients treated with surgery alone. Moreover, a pretreatment thrombocytosis status was significantly associated with higher clinical stage (odd ratio [OR]: 2.39, 95% CI: 1.68–3.38, *P* < 0.001), positive pelvic node status (OR: 1.58, 95% CI: 1.01– 2.45, *P* = 0.044) and larger tumor size (OR: 2.32, 95% CI: 1.39–3.87, *P* = 0.001). Pretreatment thrombocytosis is an independent prognosis predictor in cervical cancer patients. It may be used as a readily available biomarker to refine clinical outcome prediction for cervical cancer patients.

## INTRODUCTION

Cervical cancer is the third most normally diagnosed malignance and the fourth leading cause of cancer relevant mortality in females worldwide, with 85% of cases occurring in developing countries, and the peak incidence is in the 40–45 year age group [1]. Recently, more and more patients presenting with early stage disease is hypothesized to be the result of the increasingly widespread utilization of multi-parametric imaging, which has lead to an increase in the early detection of cervical malignance. Conventionally, radical surgery has been the mainstay treatment of early stage cervical cancer, yielding a relatively favorable prognosis [2]. However, the oncological outcome of advanced or recurrent cervical cancer remains very poor [3]. Hence, it is very important to identify prognostic factors which can be applied to predict treatment outcomes, because such predictors are helpful in guiding clinical management with respect to choosing therapy, generating suitable protocols for follow-up, and determining trial eligibility criteria. Although numerous tumor markers have been evaluated as potential prognostic predictors, such as tissue polypeptide antigen, squamous cell carcinoma antigen, cancer antigen-125, carcinoembryogenic antigen, and cytokeratin fragment 21–1 [4], it is still difficult to estimate the recurrence risk and outcome in patients with cervical cancer.

There is increasing evidence that inflammatory processes in the tumor microenvironment exert a key role during the development of several tumors [5]. As a reflection of systemic inflammatory response, pretreatment thrombocytosis, has been reported by several studies to be associated with inferior prognosis of cervical cancer. However, because of variations in results, a consensus can't be achieved. A meta-analysis published in 2012 reported thrombocytosis could predict inferior survival in cervical cancer patients [6]. Nevertheless, this study only studied overall survival (OS). Additionally, many studies embracing reliable data have been published lately. In order to obtain more detailed results, we updatedly performed a systematic review of literatures that have identified relationships of pretreatment thrombocytosis with survival and clinicopathological features in cervical cancer, and merged the extracted data to conduct a meta-analyses.

## RESULTS

### Included studies

**Figure 1:**
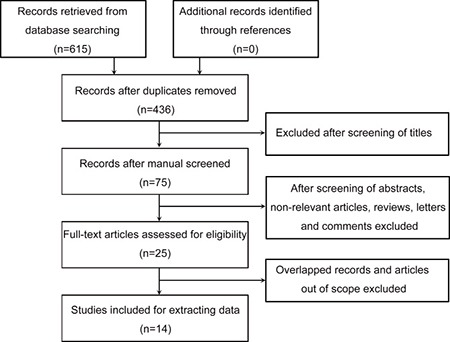
Flow chart of study selection

All of the selected studies were nonrandomized. The flow chart of the literature search was presented in Figure 1. In total, 615 relevant records were obtained from the initial literature retrieve in the above databases and no record retrieved from through references. One hundred seventy-nine duplicates were excluded from the initial records. After screening the titles of 436 studies, 75 studies were chosen for examining the abstracts. And the abstracts reviewing process recognized 25 articles, which live up to the inclusion criteria. The remaining articles were reviewed in full, finally, 14 articles were included in extracting data.

All of the included studies were published between 1992 and 2015, which comprised 3,394 patients. They had a retrospective design, with a median sample size of 219 patients (range, 46–643 patients). Most of the studies paid their attention to OS of cervical cancer patients. In 3 studies, the hazard ratio (HR) was adjusted for other relevant variables (covariates) embracing patient age, tumor size, histology, pelvic node status and clinical stage (Table 1).

**Table 1:** The main characteristics of enrolled studies

Study	Population	Study Design	Case number	Clinical stage	Treatment	Median age, y	Cut-off/nL	Survival analysis	Source of HR	Adjusted	Median follow-up, mo
Zhao_2015	China	R	220	I–IIA	Surgery	-	300	OS, RFS	SC	-	53 (6–111)
Xiao_2015	China	R	238	I–IV	RT and CT	52 (34–70)	200	OS, PFS	DE	-	42 (15–91)
Kawano_2015	Japan	R	286	I–IV	RT	63.6 ^M^	350	OS	Rep	Yes	-
Wang_2012	China	R	111	IB2-IIB	CT or surgery	42 (21–68)	266	OS, PFS	DE	-	44 (13–111)
Biedka_2012	Poland	R	53	I–IV	RT or surgery or CT-	-	-	PFS	DE	-	10 (2–31)
Qiu_2010	China	R	318	I–IV	-	43	400	OS	DE	-	-
Gadducci_2010	Italy	R	46	IB2-IIB	CT and Surgery	47 (27–70)	272	OS, RFS	SC	-	53 (4–167)
Gadducci_2010	Italy	R	140	IB2-IIB	CT and Surgery	47 (22–79)	272	OS, RFS	DE	-	56 (5–188)
Hernandez_2000	USA	R	291	IIB-IVA	Surgery and RT	50 (25–79)	400	OS	Rep	Yes	-
De Jonge_1999	South Africa	R	93	IB	Surgery	-	400	OS, RFS	Rep	Yes	45 (12–104)
Rodriguez_1994	USA	R	219	IB	Surgery	40	300	OS	DE	-	-
Lopes_1994	England	R	643	I–IV	Surgery or RT	46 (20–90)	400	OS	DE	-	-
Hernandez_1994	USA	R	623	IB	Surgery	-	400	OS, PFS	DE	-	-
Hernandez_1992	USA	R	113	I–IV	RT	59	400	OS	DE	-	-

^M^Reported as mean age.

Abbreviations: R = retrospective; RT = radiotherapy; CT = chemotherapy; OS = overall survival; DFS = disease-free survival; PFS = progression-free survival; RFS = relapse-free survival; SC = survival curve; DE = data extrapolated; Rep = Reported; –, not reported.

### Meta-analysis

**Figure 2:**
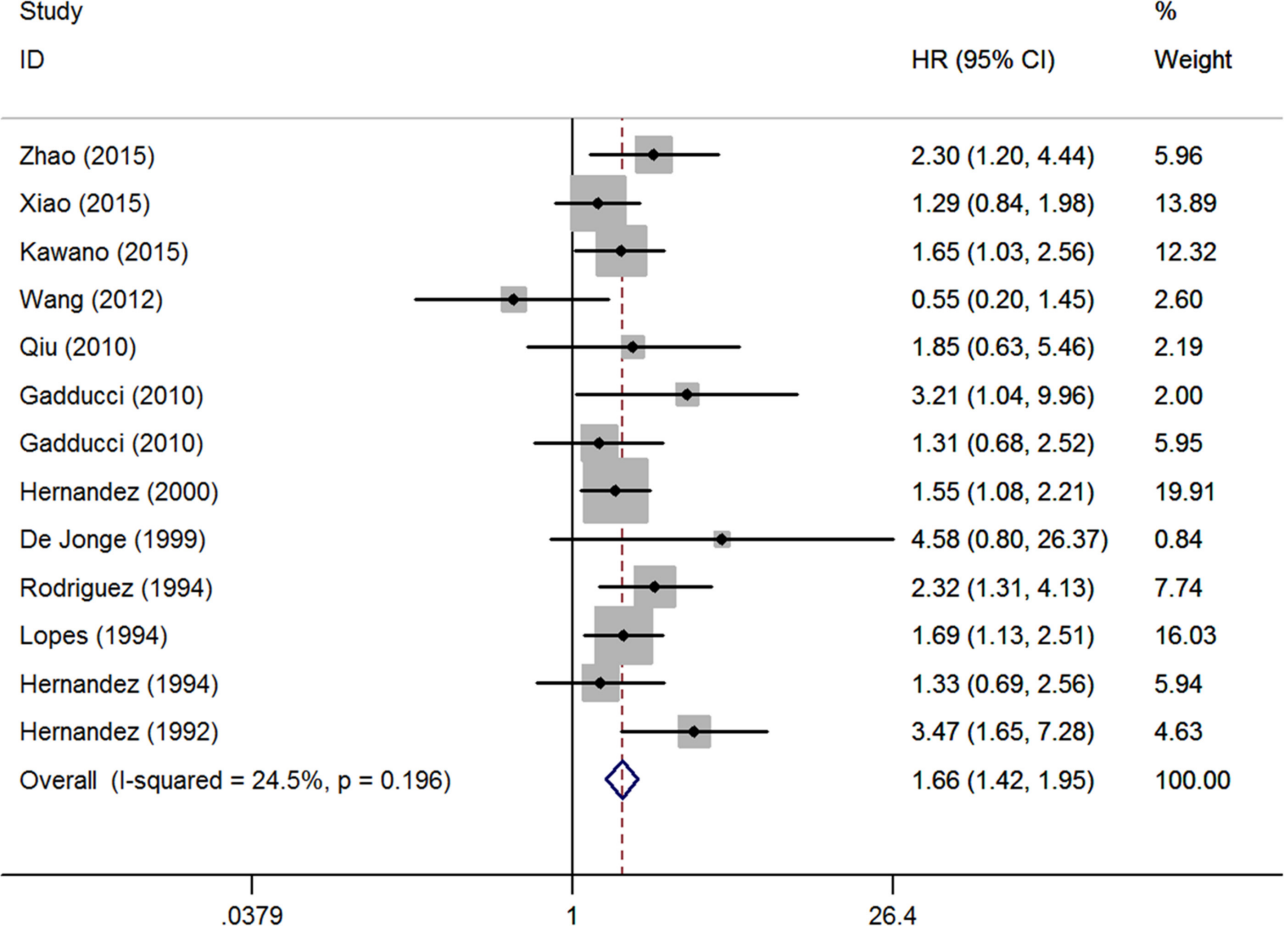
Forest plot of studies evaluating the association between pretreatment thrombocytosis and overall survival

**Figure 3:**
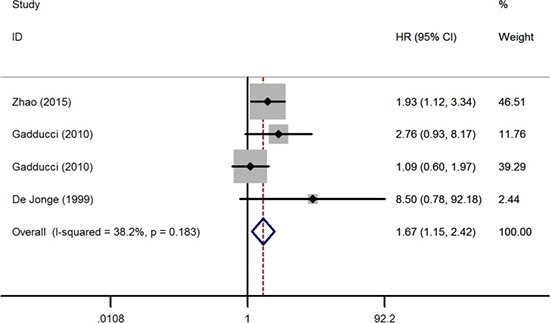
Forest plot of studies evaluating the association between pretreatment thrombocytosis and recurrence-free survival

**Figure 4:**
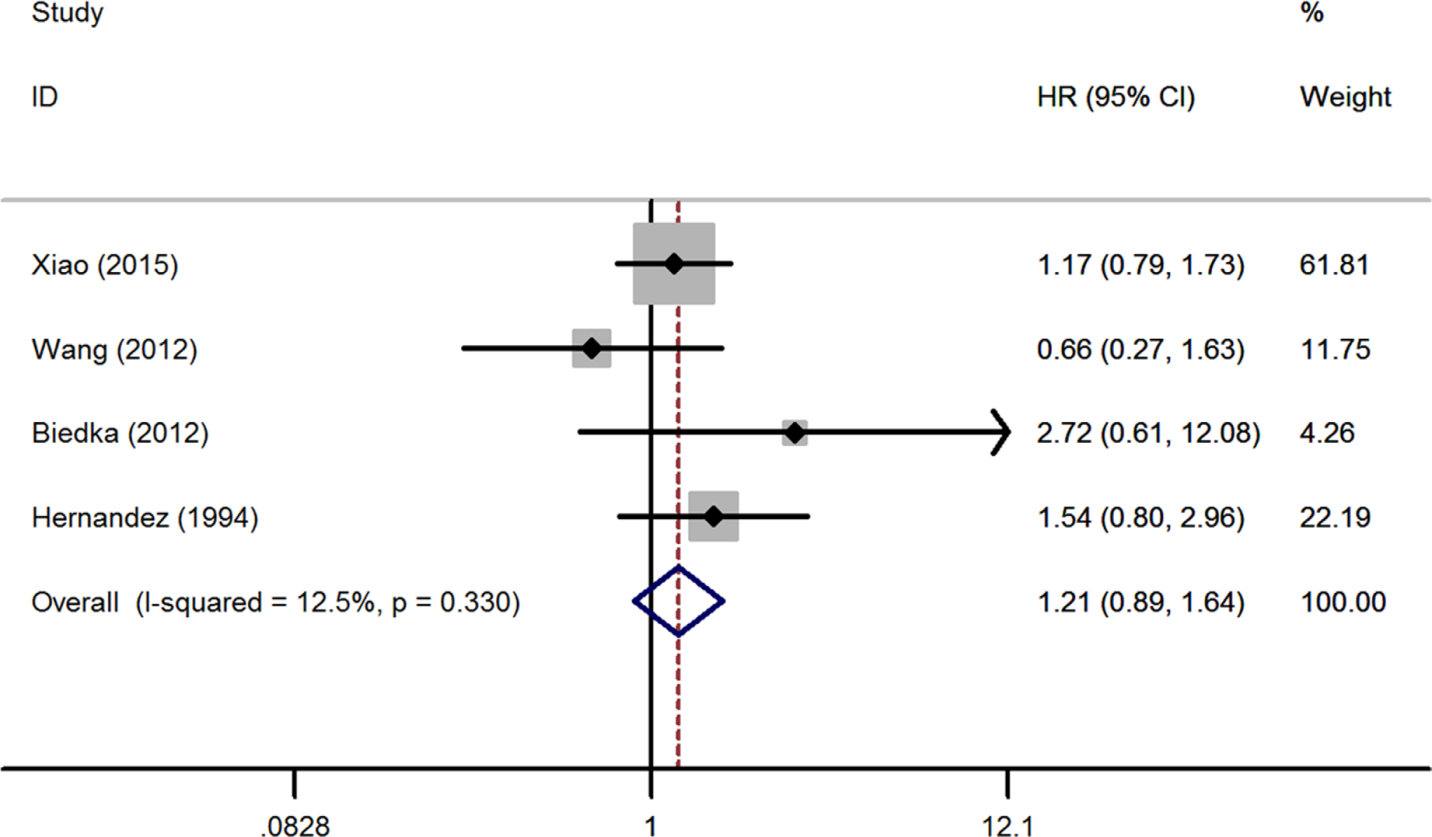
Forest plot of studies evaluating the association between pretreatment thrombocytosis and progression-free survival

At first, we studied the survival differences of cervical cancer patients presenting or absenting pretreatment thrombocytosis. The association between thrombocytosis and OS was reported in 13 studies enrolling 3,341 patients [7–19]. Six of the eligible studies reported a non-statistically significant hazard ratio, a forest plot of all studies was shown in Figure 2. A combined analysis showed that pretreatment thrombocytosis was associated with poor OS in cervical cancer (HR: 1.66, 95% CI: 1.42–1.95; *P* < 0.001). And there was absence of evidence for inter-study heterogeneity (*I*^2^ = 24.5%, *P* = 0.196). Four studies comprising 499 patients covered hazard ratios for RFS [7, 12, 13, 15], the meta-analysis found that pretreatment thrombocytosis was associated with poor RFS in cervical cancer (HR: 1.67, 95% CI: 1.15–2.42; *P* = 0.007) (Figure 3). And there was absence of evidence for inter-study heterogeneity (*I*^2^ = 38.2%, *P* = 0.183). Four studies comprising 1,025 patients reported hazard ratios for PFS [8, 10, 18, 20], meta-analyses were performed and found that the associations of pretreatment thrombocytosis with PFS (HR: 1.21, 95% CI: 0.89–1.64; *P* = 0.235) were not statistical significant (Figure 4).

Table 2 shows subgroup analyses by publication year, population, number of subjects, value of cutoff and source of HR. When stratified by these variables, there were no significant differences in pooled HRs for OS, and pretreatment thrombocytosis remained to be a significant predictor for OS.

**Table 2:** Subgroup analysis of pooled hazard ratios for OS

Studies	HR(95% CI)	*P* value	Meta-regression*P* value	Heterogeneity	
				I2 (%)	*P* value
Publication year			0.378		
1992–2000	1.80(1.45–2.23)	< 0.001		22.3	0.266
2010–2015	1.51(1.19–1.91)	0.001		27.5	0.219
Population			0.518		
Asia	1.48(1.14–1.92)	0.004		37.1	0.174
Europe	1.67(1.21–2.32)	0.002		0	0.403
Americas	1.81(1.40–2.34)	< 0.001		42.8	0.155
No. of patients			0.732		
> 150	1.64(1.37–1.95)	< 0.001		0	0.758
≤ 150	1.89(0.91–3.92)	0.087		65.3	0.021
Cut-off			0.704		
> 300	1.72(1.40–2.10)	< 0.001		0	0.468
≤ 300	1.60(1.08–2.36)	0.020		50.2	0.074
Source of HR			0.891		
univariable	1.68(1.38–2.04)	< 0.001		37.7	0.107
multivariable	1.63(1.24–2.15)	0.001		0	0.491

Abbreviations: OS = overall survival; HR = hazard ratio; CI = confidence interval.

**Figure 5:**
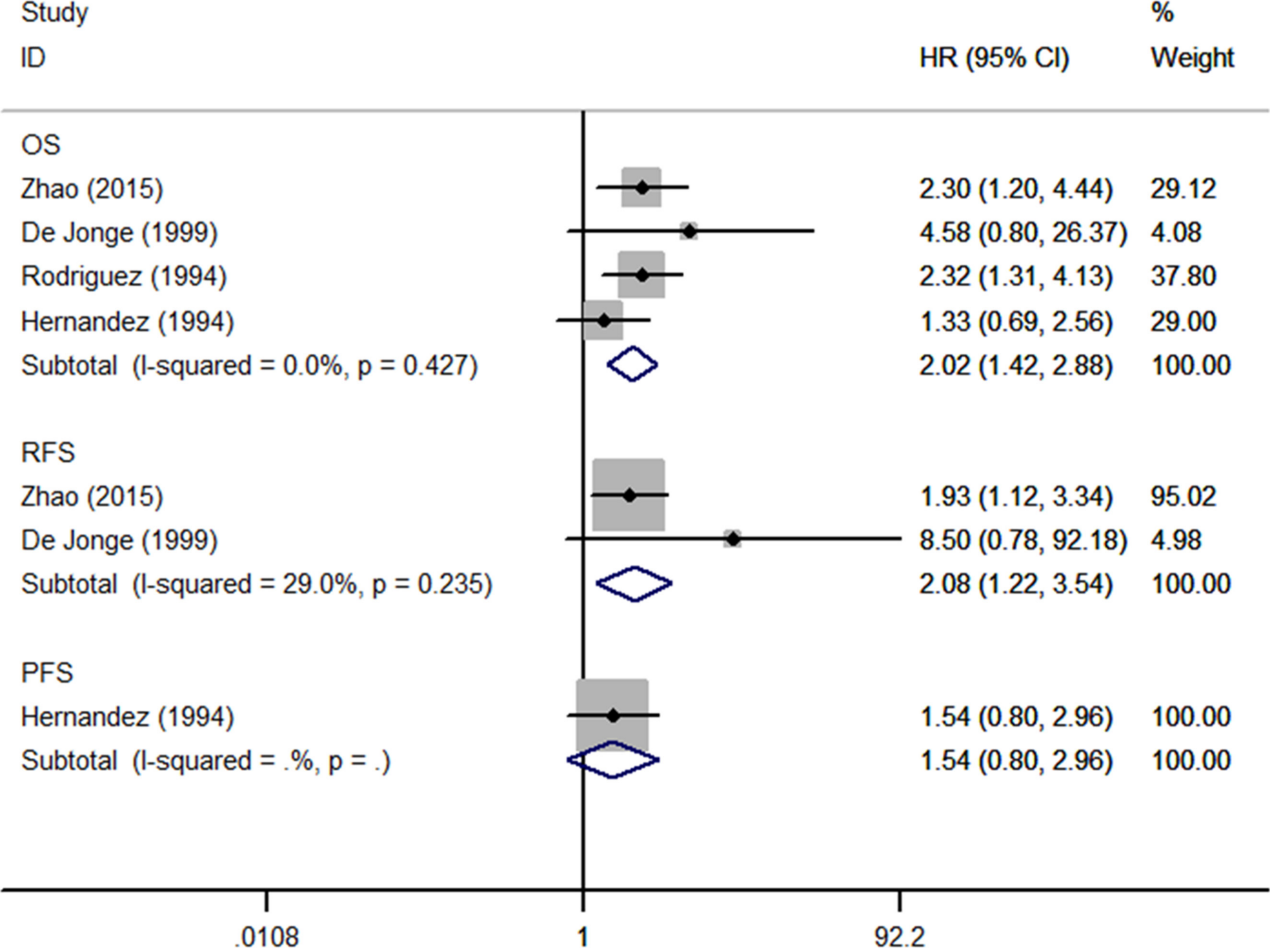
Forest plot of studies evaluated the prognostic role of pretreatment thrombocytosis in low stage patients treated with surgery alone

Four studies reported the prognostic significance of pretreatment thrombocytosis in early stage patients treated with surgery alone [7, 15, 16, 18]. A forest plot of these studies was shown in Figure 5. A combined analysis showed that pretreatment thrombocytosis was associated with inferior OS (HR: 2.02, 95% CI: 1.42–2.88; *P* < 0.001) and RFS (HR: 2.08, 95% CI: 1.22–3.54; *P* = 0.007) but not PFS (HR: 1.54, 95% CI: 0.80–2.96; *P* = 0.196) for these patients.

We then studied the relationships between thrombocytosis and the clinicopathological features of cervical cancer. Six studies provided sufficient data for the meta-analyses. As shown in Table 3, pretreatment thrombocytosis was significantly associated with clinical stage (III/IV versus I/II) (HR: 2.39, 95% CI: 1.68–3.38; *P* < 0.001), pelvic node status (positive versus negative) (HR: 1.58, 95% CI: 1.01–2.45; *P* = 0.044) and tumor size (> 4 versus ≤ 4) (HR: 2.32, 95% CI: 1.39–3.87; *P* = 0.001). These clinicopathological characteristics are indicative of poor prognosis and disease aggressiveness. We found some inter-study heterogeneity in the pelvic node status and histology (*I*^2^ = 51.9% and 78.9%, separately), whereas heterogeneity weren't exhibited in the analyses of other clinicopathological parameters (*I*^2^ = 0–33.3%).

**Table 3:** Meta-analysis of the association between thrombocytosis and clinicopathological features of cervical cancer

Variables	Studies	Patients	Pooled OR	95% CI	*P* value	Heterogeneity I^2^ (%)	*P* value
Clinical stage	3	1015	2.39	1.68–3.38	< 0.001	33.3	0.224
Pelvic node status	6	1958	1.58	1.01–2.45	0.044	51.9	0.065
Tumor size	2	460	2.32	1.39–3.87	0.001	0	0.886
Histology	4	1170	0.78	0.28–2.22	0.646	78.9	0.003

Abbreviations: OR = odds ratio; CI = confidence interval.

### Publication bias

**Figure 6:**
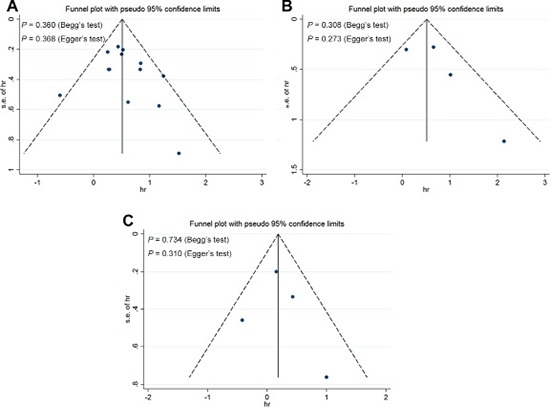
Funnel plots, Begg and Egger tests result for the evaluation of potential publication bias Plots are arranged as follows: (**A**) overall survival; (**B**) recurrence-free survival; (**C**) progression-free survival.

Publication bias were evaluated applying the funnel plot, Begg test, and Egger test in the meta-analysis. In the funnel plots, no obvious evidence of asymmetry were revealed in the contrast. Furthermore, the results from the Begg test and Egger test for the studies were *P*_Begg’s_ = 0.308–0.734 and *P*_Egger’s_ = 0.273–0.368 (Figure 6). Hence, the evidence above shows a low probability of publication bias. Sensitivity analysis suggested that excluding any single study did not significantly impact the pooled HR or OR.

## DISCUSSION

The correlation between increased platelet counts and malignancy was initial reported by Levin and Conley in 1964 [21]. In patients with malignance, particularly cases with advanced-stage tumor, tumor-related thrombocytosis occurs in 4% to 55% of them either at time of diagnosis or during the process of the disease [22]. Then, platelet count has been found as a prognosis predictor for various types of cancer [23, 24]. The thrombocytosis, reflecting the systemic inflammatory response, has been described to be correlated with adverse oncologic outcome of cervical cancer patients. Nevertheless, some studies have yielded inconsistent results. So this circumstance indicates a necessity for a systematic review and meta-analysis of the present literatures.

In the present study, the results indicated that pretreatment thrombocytosis predicts poor oncologic outcome for patients with cervical cancer, which is accordance with a previous study [6]. The results presented that patients with pretreatment thrombocytosis in cervical cancer are prone to suffer a poor OS and RFS. Nevertheless, the impact of thrombocytosis on PFS of cervical cancer patients was negligible for lacking of statistical significance. We think there are many reasons and factors causing the discordance between OS/RFS and PFS results. As we know, the outcome PFS mostly applied in high stage tumor patients. Patients have different stage tumor may have different pathophysiologic features. Moreover, the endpoints of the three outcomes are different. The mechanism of progression is different from recurrence.

Our findings also identified that cervical cancer patients with pretreatment elevated platelet count are prone to suffer a higher clinical stage, positive pelvic node metastasis and a larger tumor size, which are indicative of poor prognosis and disease aggressiveness. So platelet count is a solid prognostic factor in cervical cancer. As platelet count measurement is available and well standardized for every clinical institution, it may be used as a convenient and useful serum biomarker in supplying conventional clinicopathological variables to help clinicians to estimate patient outcome.

Our study has several advantages. First, we perform the study stringently and followed the guidelines of PRISMA, which ensured the authentic results. Second, in contrast to the previous meta-analysis [6], which was published in 2012 and embraced 5 literatures about oncologic outcomes of cervical cancer, our study included more eligible literatures to low down potential risk of bias. Third, except as OS, we also evaluated the RFS and PFS, which presented extensive evidence for the prognostic value of thrombocytosis in patients with cervical cancer. Fourth, in order to obtain more detailed results, we also performed subgroup analysis for low stage patients treated with surgery alone. As far as we know, this stands for the most comprehensive and latest review on this issue.

The reasons for the associations of elevated platelet count levels with outcome and clinicopathological features of cervical cancer as well as other solid malignancies remain uncertain. Some studies reflected that thrombocytosis was likely to be a sign of heavy tumor burden in patients, the underlying mechanism of which has been determined based on experimental evidence from several experiments [7]. Angiogenesis is a crucial step in tumor proliferation and metastasis [25]. Serving as dynamic reservoirs of various angiogenesis-regulating factors, such as VEGF, PDGF, and FGF, platelets may be conducive to tumor vascular growth [25]. Furthermore, platelet receptors and ligands can regulate tumor cell-platelet binding to modify the biological behavior of the malignance [26]. Buergy et al. [27] suggested that various growth factors and cytokines, including granulocyte-macrophage colony-stimulating factor, leukemia inhibitory factor, interleukin 1, interleukin 3, interleukin 6 (IL-6), interleukin 11, and thrombopoietin can increase the process of thrombopoiesis. A recent published study of ovarian cancer proposed that thrombocytosis might be a paraneoplastic syndrome that indicates itself through tumor-derived IL-6, which activates thrombopoiesis, resulting in thrombocytosis and tumor progression [28].

Some limitations need be taken into account in the comprehension of the results of the present study. First, each meta-analysis is obviously impacted by the quality of their constituent studies, some relevant studies lacking specific data may not be included in this meta-analysis. Because of the fact studies with non-significant or null findings were potentially less likely to be published than research with statistically significant results, pooled HRs or ORs may be overestimated as a result of reporting bias. Second, obvious inter-study heterogeneity was found in some analyses (pelvic node status and histology). Several differences among the studies could have leaded to the heterogeneity of meta-analyses, comprising patients’ baseline features (country, race, sex, age, and clinical stage and tumor size), cut-off values for platelet count, treatment strategy for patients, and length of follow-up. Because of methodologic limitations of the non-randomized studies included, the results might be affected by some potential unmeasured or residual confounders. Additionally, the method of extracting the HRs and 95% CI from included literatures was also a possible reason that could have result in heterogeneity. Of the 14 studies, 3 directly provided HRs, and the methods described by Tierney et al. [29] were used to calculate individual HRs of the left literatures. The calculated HRs might be not as dependable as those obtained from statistics reported directly. Third, because of limited literatures, most of meta-analyses included a few studies, which might unavoidably raise the risk of random error. Therefore, additional well-designed and appropriately conducted prospective research are needed to verify a more persuasive association between thrombocytosis and cervical cancer.

Despite the limitations described above, our comprehensive systematic review and meta-analysis reveals that pretreatment thrombocytosis is significantly associated with inferior OS and RFS in cervical cancer patients. It may be used as a readily available biomarker supplying conventional clinicopathological variables to refine clinical outcome prediction for cervical cancer patients, though large prospective studies are needed to demonstrate our findings.

## MATERIALS AND METHODS

This meta-analysis was performed in the light of the guidelines of Preferred Reporting Items for Systematic Reviews and Meta-Analyses (PRISMA) [30].

### Search strategy

A systematic literature search was conducted in June 2016 using PubMed, Embase, Web of Science databases and the Cochrane Library. The searches were not limited by language. Our search strategy included terms for: “thrombocytosis” (e.g., “thrombocytosis,” “thrombocythemia,” “platelet count,” “platelet”), “prognosis” (e.g., “prognosis,” “outcome,” “survival,” “mortality,” “recurrence” “progression,” “metastasis”) and “cervical cancer” (e.g., “cervical cancer,” “cervical tumor,” “cervical neoplasm” “cervical carcinoma”). In addition, the references from the related literatures embracing the all retrieved studies, editorials, and reviews were manually screened.

### Study selection

Inclusion criteria for selecting the articles for our analysis were as follows: (1) retrospective or prospective study design; (2) studies of people with cervical cancer reporting on the prognostic impact of the peripheral blood platelet count; (3) treatment limited to surveillance, surgery, radiotherapy, or chemotherapy; (4) measurement of platelet count before specific treatments; (5) clearly described outcome evaluation containing overall survival (OS), recurrence-free survival (RFS) or progression-free survival (PFS); (6) oncologic outcome was further investigated regarding hazard ratio (HR) with confidence interval (CI), HR with *P value*, Kaplan-Meier curves or the needed data for calculating HR and CI; and (7) median follow-up longer than 6 months.

Exclusion criteria were as follows: (1) non-human research; (2) were editorials, letters, case reports, expert opinions, reviews, meeting records; (3) post-treatment platelet count; (4) didn't analyzed platelet count as a dichotomized variable; or (5) lacked competent data for calculating HRs and their 95% CIs. When more than one studies analyzing the same patient cohort were retrieved, we selected the more well-designed, recent and informative publication. Two individual researchers (J.C and Z.Z) independently assessed eligibility of the retrieved articles. The disagreements were resolved through discussion.

### Data extraction and synthesis

OS was the primary outcome of interest. RFS and PFS were secondary outcomes. OS was deemed to the period between initiation of treatment and all-causes death or censored. RFS was calculated as time between initiation of treatment and the date of recurrence or censored. PFS was deemed to time between initiation of treatment and the date of progression or censored. Data were extracted independently by two investigators (J.C and Z.Z), who used a predefined sheet to regain information from all studies that certified for final inclusion. We designed data sheets according to former literatures concerning similar topic and PRISMA guideline [24, 30]. The following information were extracted: (1) publication information: first author's last name, publication year and design of study; (2) patients’ characteristics: population study, number of patients, clinical stage of cancer, treatment pattern, median age of patients; (3) cut-off value; and (4) HR and 95% CI for OS, RFS or PFS as applicable. If available, HRs with their 95% CIs were extracted preferentially from multivariable analyses. If not, HRs and their 95% CIs from univariate analyses were extracted. If only Kaplan-Meier curves were available, we extracted data from the graphical survival plots and estimated the HRs and 95% CIs. Or HRs and 95% CIs were calculated using the data of observed events, the data of samples in each group or the data provided by the authors. All the calculations mentioned above were based on the methods described by Tierney et al. [29].

The relationships between thrombocytosis and the clinicopathological features of cervical cancer patients were also studied. We dichotomized data about clinical stage (III/IV versus I/II), pelvic node status (positive versus negative), tumor size (> 4 versus ≤ 4), and histology (SCC (squamous cell carcinoma) versus non-SCC). Original data were extracted from included studies to calculate the odds ratio (OR) and corresponding 95% CI.

### Statistical analysis

Cochran's *Q* test and Higgins *I*-squared statistic were used to identify heterogeneity of combined HRs and ORs. A *P value* < 0.1 was considered significant. *I*^2^ more than 50% is considered as a measure of severe heterogeneity. When heterogeneity was obvious, a random-effect model was applied. If not, we applied a fixed-effect model. An observed HR or OR > 1 indicated inferior oncologic outcome for patients with thrombocytosis or a significant association between thrombocytosis and patients features. We used funnel plot visual inspection, Begg (rank correlation analysis) and Egger tests (linear regression analysis) to assess publication bias. Sensitivity analysis was also conducted by deletion of each single study to assess stability of the results. Subgroup analysis and meta-regression were performed for OS. All analyses were conducted with Stata 12.0 software (StatCorp, College Station, TX, USA). All statistical tests were two-sided, and statistical significance was deemed to *P* less than 0.05.
